# Relationship between serum sodium levels and all-cause mortality in congestive heart failure patients: A retrospective cohort study based on the Mimic-III database

**DOI:** 10.3389/fcvm.2022.1082845

**Published:** 2023-01-13

**Authors:** Shixuan Peng, Jianxing Peng, Lianju Yang, Weiqi Ke

**Affiliations:** ^1^Department of Oncology, Graduate Collaborative Training Base of The First People's Hospital of Xiangtan City, Hengyang Medical School, University of South China, Hengyang, Hunan, China; ^2^Department of Orthopaedics, Anxiang People's Hospital, Changde, Hunan, China; ^3^Department of Health Management Centre, Anxiang People's Hospital, Changde, Hunan, China; ^4^Department of Anesthesiology, The First Affiliated Hospital of Shantou University Medical College, Shantou, Guangdong, China

**Keywords:** congestive heart failure, serum sodium levels, Cox proportional hazards regression, all-cause mortality, heart failure

## Abstract

**Background:**

The relationship between serum sodium levels and mortality in congestive heart failure (CHF) patients has not been well-studied previously. The non-linear correlation between serum sodium levels and mortality in patients with heart failure is currently controversial, and the relationship between different serum sodium levels and mortality is disputed. The goal of this study is to look into the relationship between serum sodium levels and all-cause mortality in people with CHF after controlling for other factors.

**Methods:**

The publicly accessible Mimic III database was the source of data for our study. We use the ICU Admission Scoring System to collect demographic data, laboratory findings, comorbidities, vital signs, and scoring information for each patient. Cox proportional risk analysis, smooth curve fitting, and the Kaplan-Meier survival curve were used to assess the relationship between baseline sodium levels and all-cause mortality in CHF patients.

**Results:**

The segmentation regression model discovered a turning point value of serum sodium levels (137.5 mmol/L) between serum sodium levels and all-cause mortality. According to the results of the fully adjusted Cox proportional hazard model, lower serum sodium levels (<137.5 mmol/L) were associated with an increased risk of 30, 90, 365-day, and 4-year all-cause deaths. The HRs and 95th confidence intervals were 0.96 (0.94, 0.99), 0.96 (0.94, 0.99), 0.96 (0.94, 0.98), and 0.96 (0.95, 0.98), respectively; the higher serum sodium levels (≥137.5 mmol/L) were related to an associated multiplied risk of 30, 90, 365-day, and 4-year all-cause deaths; the HRs and 95th confidence intervals were 1.02 (1.00, 1.05), 1.02 (1.00, 1.04), 1.02 (1.00, 1.03), and 1.02 (1.00, 1.03), respectively.

**Conclusion:**

Serum sodium levels were u-shaped about all-cause mortality. In individuals with CHF, serum sodium levels are linked to an elevated risk of short-, medium-, and long-term all-cause mortality.

## Introduction

Serum sodium refers to the concentration of sodium ions in serum. The sodium ion is the most abundant cation in extracellular fluids (e.g., blood). It is crucial for maintaining proper osmotic pressure, sustaining cellular physiological processes, maintaining extracellular fluid levels, and controlling acid-base balance (1). The renin-angiotensin-aldosterone system (RAAS), antidiuretic hormone activation, and sympathetic nerve stimulation are the main causes of heart failure. Adequate cardiac output can still be maintained through sodium and water retention and improved myocardial contraction, but these mechanisms will eventually lead to direct cytotoxicity, causing myocardial fibrosis, proarrhythmia, and pump failure. Congestive heart failure (CHF) patients' outcomes are known to be predicted by their serum sodium levels (2). In earlier research, it was also demonstrated to predict death in patients hospitalized for heart failure. However, we failed to discover any reliable research to support a relationship between serum sodium levels and long-term mortality in heart failure patients.

Serum sodium levels are an independent predictor of progressive heart failure, and among outpatients with chronic heart failure, patients with low blood sodium have an increased risk of death, with a 2 mmol/l reduction in serum sodium levels and a hazard ratio of 1.22 (95% CI 1.08–1.38) (3). Hyponatremia and hypotension are associated with an increased risk of death from heart failure (4). A change in serum sodium concentration over time among patients hospitalized with heart failure and hyponatraemia was demonstrated by Madan et al. (5).

Lack of sodium and water can result in pulmonary or peripheral oedema, which increases the risk of morbidity and mortality in individuals with heart failure. The clinical syndrome of congestive heart failure is defined by this combination of an excess of total body fluid volume and ventricular dysfunction (6). The non-linear correlation between serum sodium levels and mortality in patients with heart failure is currently controversial, and the relationship between different serum sodium levels and mortality is disputed. The goal of the current study was to determine whether serum sodium levels are independently related to 30, 90, 365, and 4-year mortality in patients with heart failure, in order to investigate this disease deeply.

### Participants and methods

#### Study design

This study used a retrospective cohort design to investigate the relationship between serum sodium levels and all-cause mortality. The target-independent variable is the serum sodium level obtained at baseline. The dependent variables are 30, 90, 365, and 4-year all-cause deaths (dichotomous variable: 1= death; 0 = survival).

#### Study population

A total of 5,002 individuals were chosen for the study, with a male preponderance of 53.2% and an average age of 72.4 ± 13.5 years. The patients with congestive heart failure in our study were enrolled from 2001-1 to 2008-12. The MIT Computational Physiology Laboratory, Beth Israel Deacon Medical Center, and Philips Medical created the Multiparameter Intelligent Monitoring in Intensive Care III Version 1.4 (Mimic-III V. 1.4) database. More than 50,000 patients who were admitted to the intensive care unit at Beth Israel Deacon Medical Center between 2001 and 2012 have clinical data available in this free and public database (7). We successfully gathered information on 8,952 patients with congestive heart failure using PostgreSQL Structured Query Language based on International Classification of Diseases (ICD-9) codes.

#### Inclusion and exclusion criteria

The prerequisites for participation were as follows: (1) Patients in the open-source MIMIC-III database (more than 50,000 patients) with the ICD-9 disease codes 4280-4289 and 39,891 (CHF patients); (2) Patients with a first admission and those with a first admission to the ICU (*n* = 8,952).

The following restrictions were in place: Patients with the following characteristics are excluded: (1) those under the age of 18; (2) those with an ICU stay of fewer than 24 h; (3) those with leukemia and myelodysplastic syndrome; (4) those with Dbsource = metavision; and (5) those with missing baseline serum sodium values at ICU admission.

#### Variables

The study's initial serum sodium levels were measured and recorded as a continuous variable. As a dichotomous variable, the all-cause death during the course of 30, 90, 365 days, and 4 years was tracked.

We obtained the ultimate outcome variable in accordance with research and guidelines that have been published (dichotomous variable). This analysis considered the following covariables as factors to consider: (1) demographic data; (2) vital signs; (3) laboratory parameters; and (4) comorbidities.

As a result, the fully adjusted model was developed using the variables below: (1) continuous variables (obtained at baseline): age; heart rate; systolic blood pressure (SBP); temperature; pulse oxygen saturation (SPO_2_); diastolic blood pressure (DBP); respiratory rate; albumin level; blood urea nitrogen (Bun) level; sodium level; prothrombin time (Pt); platelet level; hemoglobin level; partial thromboplastin time (Ptt); hematocrit level; red blood cell (RBC) count; glucose level; potassium level; creatinine level; bicarbonate level; serum anion gap; white blood cells (WBC) count; red blood cell distribution width (RDW) level; and (2) categorical variables (obtained at baseline): sex; admission type; insurance type; deficiency anemias; blood loss anemia; coagulopathy; renal failure; hypothyroidism; complicated diabetes; uncomplicated diabetes; peripheral vascular disease; hypertension; liver disease; pulmonary circulation; valvular disease; chronic pulmonary disease; and cardiac arrhythmias.

## Statistical analysis

Categorical variables are expressed as frequencies and percentages, while continuous variables are expressed as mean standard deviation (SD) (Gaussian distribution) or median (min, max) (skewed distribution). The demographics, baseline serum sodium levels, and clinical characteristics were compared among groups of patients by dividing the normal serum sodium level reference range (135–145 mmol/L) into four intervals. We divided serum sodium levels into four intervals based on the normal reference range for serum sodium: one interval containing patients with a serum sodium below 135 mmol/L, one with patients with a serum sodium from 135 to 140 mmol/L, one with patients with a serum sodium from 140 to 145 mmol/L, and lastly, one interval containing patients with a serum sodium above 145 mmol/L. Thus, this study contains 4 serum sodium intervals, where the reference interval was defined as serum sodium from 135 to 140 mmol/L based on analysis, which confirmed that the lowest mortality risk was found in this range (Table 1). Categorical variables are expressed as counts and percentages, and continuous values are expressed as medians and interquartile ranges. We used Pearson's χ2 test to assess differences in categorical variables and the Kruskal-Wallis rank sum test to assess differences in non-normally distributed continuous variables (8). The analysis of serum sodium includes continuous variables, dichotomous classification, and quadruple classification. We created three different models using univariate and multivariate Cox proportional-hazard regression models, including an unadjusted model (unadjusted covariates), a minimally adjusted model (sociodemographic characteristics were the only covariate considered), and a fully adjusted model (factors listed in Table 1 were adjusted) to examine the relationship between serum sodium concentration and deaths from all causes. Include a 95% confidence interval alongside the effect size. Non-linearity between serum sodium levels and all-cause mortality was resolved by a Cox proportional hazards regression model using a cubic spline function and smooth curve fitting, despite methods based on the Cox proportional-hazards regression model frequently being suspected of being unable to handle non-linear models (the penalized spline method). After non-linearity was discovered, the turning point was initially determined using a recursive approach, and on both sides of the turning point, a two-part Cox proportional hazards regression model was built. Using Kaplan-Meier curves, differences in mortality between patients in each group with various serum sodium levels at entry were evaluated. The proportional risk assumption is satisfied if there is no crossover between the K-M survival curves in each group. We conducted a sensitivity analysis to evaluate the robustness of the results. To confirm serum sodium levels as a result of the continuous variable and test for non-linearity, we converted serum sodium levels into a categorical variable in accordance with the bisection and calculated the trend of P.

**Table 1:** Participant's baseline characteristics (*N* = 5,002).

**Sodium (mmol/L)groups**	**Total**	**G1 (< 135)**	**G2 (≥135, < 140)**	**G3 (≥140, < 145)**	**G4 (≥145)**	***P*-value**
Number, *n*	5,002	820	2,424	1,482	276	
Age (years)	72.4 ± 13.5	71.1 ± 13.7	71.4 ± 13.0	73.6 ± 14.0	78.1 ± 11.4	< 0.001
Gender, *n* (%)						< 0.001
Male	2,662 (53.2%)	428 (52.2%)	1,364 (56.3%)	747 (50.4%)	123 (44.6%)	
Female	2,340 (46.8%)	392 (47.8%)	1,060 (43.7%)	735 (49.6%)	153 (55.4%)	
**Admission type**, ***n*** **(%)**						< 0.001
Emergency	4,030 (80.6%)	688 (83.9%)	1,849 (76.3%)	1,236 (83.4%)	257 (93.1%)	
Elective	793 (15.9%)	86 (10.5%)	486 (20.0%)	208 (14.0%)	13 (4.7%)	
Urgent	179 (3.6%)	46 (5.6%)	89 (3.7%)	38 (2.6%)	6 (2.2%)	
**Insurance**, ***n*** **(%)**						< 0.001
Medicare	3,694 (73.9%)	596 (72.7%)	1,728 (71.3%)	1,134 (76.5%)	236 (85.5%)	
Private	989 (19.8%)	155 (18.9%)	539 (22.2%)	268 (18.1%)	27 (9.8%)	
Medicaid	237 (4.7%)	49 (6.0%)	120 (5.0%)	57 (3.8%)	11 (4.0%)	
Government	61 (1.2%)	12 (1.5%)	30 (1.2%)	18 (1.2%)	1 (0.4%)	
Self Pay	21 (0.4%)	8 (1.0%)	7 (0.3%)	5 (0.3%)	1 (0.4%)	
**Vital signs**						
Heart rate (bpm)	85.3 ± 15.4	85.8 ± 15.8	85.5 ± 14.9	84.5 ± 16.0	87.0 ± 16.3	0.038
SBP (mmHg)	116.8 ± 16.9	113.5 ± 17.1	116.0 ± 16.1	119.5 ± 17.5	120.3 ± 16.8	< 0.001
DBP (mmHg)	56.8 ± 9.9	55.9 ± 9.4	56.7 ± 9.6	57.6 ± 10.4	56.4 ± 10.1	< 0.001
respiratory rate (bpm)	19.4 ± 4.1	19.4 ± 4.1	19.1 ± 4.0	19.7 ± 4.2	20.7 ± 4.8	< 0.001
Temperature (°C)	36.8 ± 0.6	36.8 ± 0.7	36.9 ± 0.6	36.9 ± 0.6	36.9 ± 0.7	0.004
SPO_2_ (%)	97.1 ± 2.2	97.1 ± 2.3	97.2 ± 2.1	97.0 ± 2.3	96.9 ± 2.4	0.011
**Laboratory parameters**						
Albumin (g/dl)	3.1 ± 0.6	3.0 ± 0.6	3.2 ± 0.6	3.2 ± 0.6	2.9 ± 0.6	< 0.001
Anion gap (mmol/L)	14.8 ± 3.4	15.4 ± 4.0	14.6 ± 3.4	14.6 ± 3.2	14.8 ± 3.4	< 0.001
Bicarbonate (mmol/L)	24.1 ± 4.7	23.0 ± 4.6	23.8 ± 4.2	24.9 ± 5.1	25.3 ± 6.3	< 0.001
Creatinine (mEq/L)	1.6 ± 1.5	1.9 ± 1.8	1.6 ± 1.6	1.5 ± 1.4	1.6 ± 1.0	< 0.001
Glucose (mg/dL)	150.2 ± 53.2	158.1 ± 68.0	151.3 ± 51.2	144.1 ± 45.7	150.6 ± 54.1	< 0.001
Hematocrit (%)	32.2 ± 5.1	31.4 ± 4.7	32.1 ± 5.1	32.8 ± 5.4	32.6 ± 4.7	< 0.001
Hemoglobin (g/dL)	10.8 ± 1.8	10.6 ± 1.7	10.8 ± 1.8	11.0 ± 1.9	10.7 ± 1.6	< 0.001
Platelet (10^9^/L)	220.2 ± 105.4	222.8 ± 111.6	217.6 ± 102.7	222.1 ± 105.4	226.1 ± 110.0	0.346
Sodium (mmol/L)	138.4 ± 4.3	131.9 ± 3.1	137.5 ± 1.4	141.7 ± 1.3	147.5 ± 3.2	< 0.001
Potassium (mmol/L)	4.3 ± 0.6	4.4 ± 0.6	4.3 ± 0.5	4.1 ± 0.5	4.0 ± 0.6	< 0.001
Ptt (seconds)	42.2 ± 21.3	43.9 ± 20.4	42.4 ± 20.8	41.5 ± 22.5	38.9 ± 21.1	0.006
Pt (seconds)	15.9 ± 5.7	16.6 ± 7.4	15.7 ± 4.9	15.8 ± 5.6	16.4 ± 6.6	< 0.001
Bun (mg/dL)	32.6 ± 23.1	36.8 ± 26.9	30.3 ± 22.1	31.9 ± 21.1	43.5 ± 25.0	< 0.001
WBC (10^9^/L)	12.8 ± 10.3	13.2 ± 11.1	12.7 ± 5.9	12.5 ± 14.6	14.0 ± 10.9	0.078
RDW (%)	15.2 ± 1.9	15.5 ± 2.2	15.1 ± 1.8	15.2 ± 1.9	15.7 ± 2.2	< 0.001
RBC (10^12^/L)	3.6 ± 0.6	3.5 ± 0.6	3.6 ± 0.6	3.7 ± 0.6	3.6 ± 0.5	< 0.001
SOFA	4.9 ± 2.9	5.4 ± 3.3	4.8 ± 2.9	4.6 ± 2.8	5.2 ± 2.8	< 0.001
SAPSII	39.7 ± 12.9	42.3 ± 13.6	38.3 ± 12.7	39.3 ± 12.6	45.9 ± 12.0	< 0.001
EVCI	8.0 ± 7.0	8.8 ± 7.3	7.0 ± 6.9	8.4 ± 6.8	12.4 ± 6.2	< 0.001
**Comorbidities**, ***n*** **(%)**						
Cardiac arrhythmias	1,185 (23.7%)	178 (21.7%)	462 (19.1%)	433 (29.2%)	112 (40.6%)	< 0.001
Valvular disease	466 (9.3%)	81 (9.9%)	183 (7.5%)	166 (11.2%)	36 (13.0%)	< 0.001
Pulmonary circulation	184 (3.7%)	32 (3.9%)	72 (3.0%)	64 (4.3%)	16 (5.8%)	0.033
Peripheral vascular	559 (11.2%)	102 (12.4%)	291 (12.0%)	141 (9.5%)	25 (9.1%)	0.039
Hypertension	674 (13.5%)	132 (16.1%)	326 (13.4%)	181 (12.2%)	35 (12.7%)	0.072
Chronic pulmonary	1,210 (24.2%)	183 (22.3%)	563 (23.2%)	388 (26.2%)	76 (27.5%)	0.053
Diabetes uncomplicated	1,213 (24.3%)	195 (23.8%)	626 (25.8%)	342 (23.1%)	50 (18.1%)	0.017
Diabetes complicated	443 (8.9%)	86 (10.5%)	231 (9.5%)	102 (6.9%)	24 (8.7%)	0.011
Hypothyroidism	460 (9.2%)	76 (9.3%)	211 (8.7%)	148 (10.0%)	25 (9.1%)	0.61
Renal failure	879 (17.6%)	169 (20.6%)	421 (17.4%)	242 (16.3%)	47 (17.0%)	0.074
Liver disease	173 (3.5%)	47 (5.7%)	90 (3.7%)	29 (2.0%)	7 (2.5%)	< 0.001
Coagulopathy	560 (11.2%)	109 (13.3%)	244 (10.1%)	172 (11.6%)	35 (12.7%)	0.055
Blood loss anemia	120 (2.4%)	23 (2.8%)	46 (1.9%)	45 (3.0%)	6 (2.2%)	0.121
Deficiency anemias	905 (18.1%)	160 (19.5%)	425 (17.5%)	269 (18.2%)	51 (18.5%)	0.646
30-day mortality, n (%)	943 (18.9%)	187 (22.8%)	366 (15.1%)	291 (19.6%)	99 (35.9%)	< 0.001
90-day mortality, n (%)	1,335 (26.7%)	261 (31.8%)	533 (22.0%)	415 (28.0%)	126 (45.7%)	< 0.001
365-day mortality, n (%)	1,910 (38.2%)	371 (45.2%)	792 (32.7%)	585 (39.5%)	162 (58.7%)	< 0.001
4-year mortality, n (%)	2,778 (55.5%)	501 (61.1%)	1,212 (50.0%)	851 (57.4%)	214 (77.5%)	< 0.001

SBP, Systolic blood pressure; RBC, red blood cell; PTT, partial thromboplastin time; BUN, blood urea nitrogen; WBC, white blood cell; PT, prothrombin time; RDW, Red Blood Cell Distribution Width; DBP, Diastolic blood pressure; SAPSII, simplified acute physiology score II; EVCI, Elixhauser-van Walraven Comorbidity Index; SOFA, sequential organ failure assessment.

The statistical tools R (http://www.r-project.org, The R Foundation) and EmpowerStats (http://www.empowerstats.com, X&Y Solutions, Inc., Boston, MA) were used to conduct the statistical tests. Significant values were defined as *p* < 0.05 (9).

## Results

### Characteristics and clinical parameters of CHF patients

**Figure 1:**
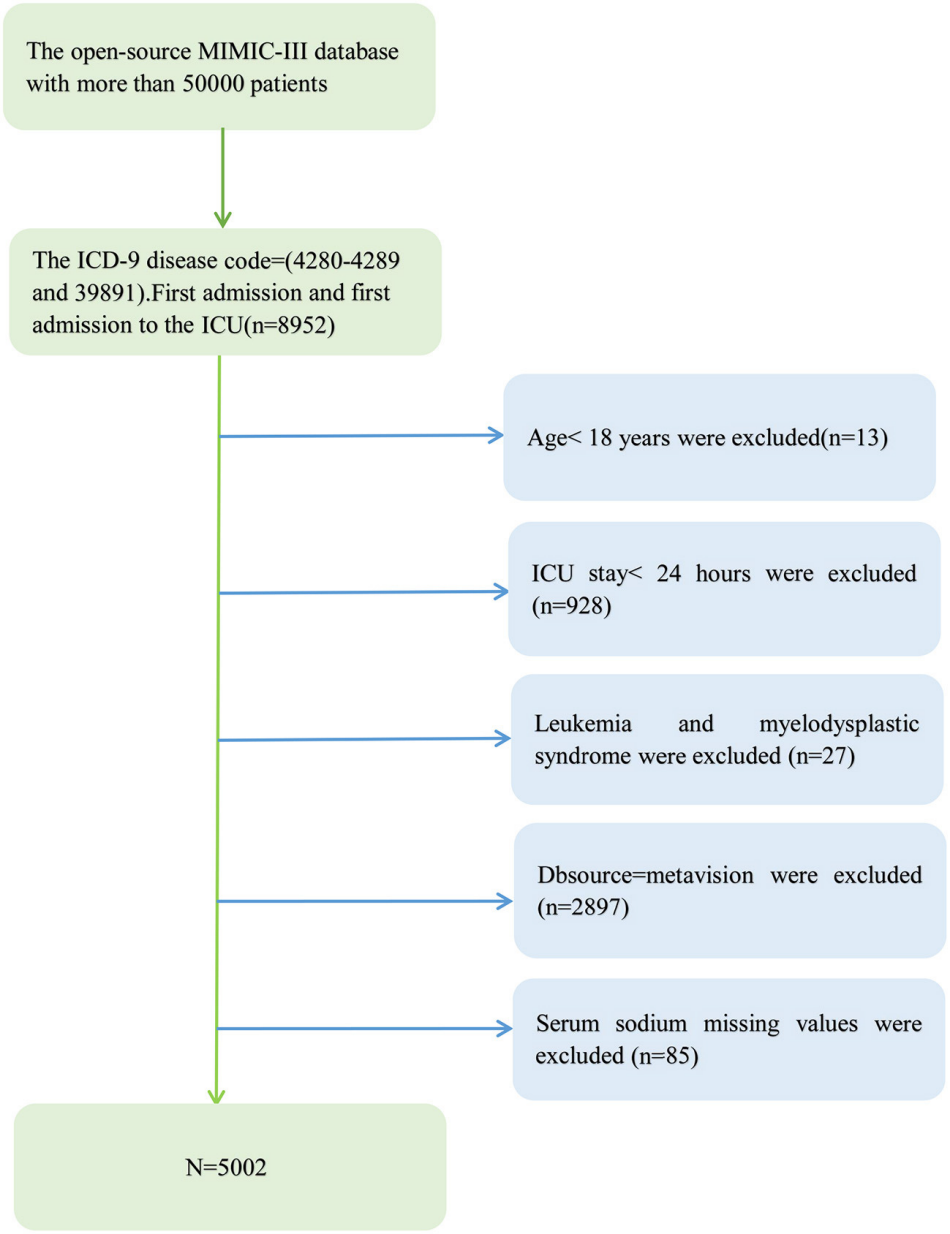
Flowchart of patient selection.

**Figure 2:**
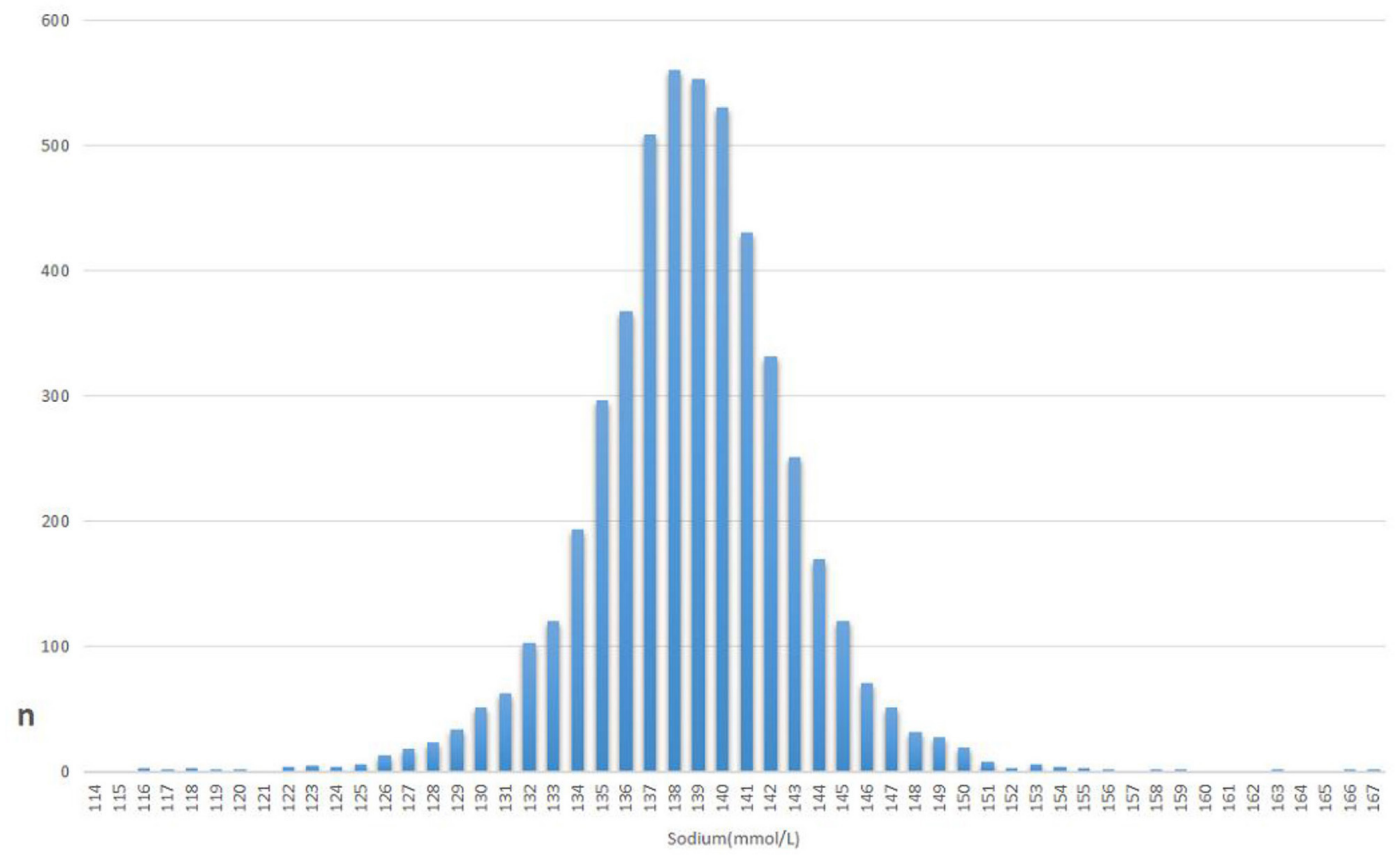
The histogram of the number of patients and the serum sodium levels.

After screening according to eligibility requirements, 5,002 patients were selected for the final analysis of the data (Figure 1). Table 1 lists the initial features of these chosen participants in accordance with the sodium group's crucial points. Overall, the mean age of the 5,002 selected participants was 72.4 ± 13.5 years, of whom ~53.2% were male. There are no statistically significant differences in platelets, WBC, Hypertension, Chronic Pulmonary Disease, Hypothyroidism, Renal Failure, Coagulopathy, Blood Loss Anemia, or Deficiency Anemia between sodium (mmol/L) groups (all *p* > 0.05). The histogram of the number of patients and the serum levels shows the concentration trend is a normal distribution (Figure 2).

Participants with the lowest group of sodium (mmol/L) groups [Sodium (mmol/L) groups<135] had the higher values in Anion gap, Creatinine, Glucose, Potassium, ptt, pt, SOFA, and the lower in SBP, DBP, Bicarbonateand, Hemoglobin, Hematocrit, RBC, this group consisted of more patients with Peripheral vascular disease, hypertension, Diabetes complicated, Renal failure, Liver disease, Coagulopathy, Deficiency anemias in contrast with those in the other groups.

Participants with the group of Sodium (≥135, <140 mmol/L) had higher values in SPO_2_, and lower values in respiratory rate, EVCI, Platelet, Pt, RDW, and Bun, this group consisted of more patients with Diabetes uncomplicated in contrast with those in the other groups.

Participants with the group of Sodium (≥140, <145 mmol/L) had higher values in Hematocrit, Hemoglobin, and lower values in Heart rate, Anion gap, Creatinine, Glucose, WBC, and SOFA, this group consisted of more patients with Hypothyroidism, Blood loss anemia in contrast with those in the other groups.

Participants with the highest group of Sodium (mmol/L) groups (Sodium (mmol/L) groups≥145) had higher values in SBP, respiratory rate, Temperature, Hematocrit, Platelet, Bun, WBC, RDW, SOFA, SAPS II, EVCI, and the lower values in DBP, SPO_2_, Albumin, Bicarbonate Potassium, and Ptt; this group consisted of more patients with Pulmonary circulation, Cardiac arrhythmias, Chronic pulmonary, Valvular disease in contrast with those in the other groups. Most of the CHF patients in this group were older.

### Threshold effect analysis for the relationship between serum sodium levels and all-cause mortality

By spline smoothing and adjusting for other factors, the link between serum sodium levels and all-cause mortality was found to be U-shaped, with the 95% confidence interval from smoothing represented by imaginary lines. A turning point value of serum sodium levels (137.5 mmol/L) was found by a segmentation regression model between serum sodium levels and all-cause mortality. The mortality rate of CHF patients was lowest when the serum sodium levels were 137.5 mmol/L. It shows that serum sodium levels were related to associate multiplied risk of 30, 90, 365, and 4-year all-cause deaths, and when serum sodium levels ≥137.5 mmol/L, all-cause mortality increased, respectively by 3, 3, 3, 2% in the CHF patients per unit increase of serum sodium levels (adjusted HR = 1.03, 95%CIs: 1.01, 1.06; HR = 1.03, 95%CIs: 1.01, 1.05; HR = 1.03, 95%CIs: 1.01, 1.04; HR = 1.02, 95%CIs: 1.01, 1.04); When serum sodium levels<137.5 mmol/L, all-cause mortality decreased, respectively by 5, 5, 5, 4% per unit increase of serum sodium levels (adjusted HR = 0.95, 95%CIs: 0.92, 0.98; HR = 0.95, 95%CIs: 0.93, 0.98; HR = 0.95, 95%CIs: 0.93, 0.97; HR = 0.96, 95%CIs: 0.94, 0.98) (LRT test: *P* < 0.001, indicating a non-linear association between serum sodium levels and all-cause mortality) (Table 2).

**Table 2:** Threshold effect analysis for the relationship between serum sodium levels and all-cause mortality.

**Outcome**	**30-day mortality**	**90-day mortality**	**365-day mortality**	**4-year mortality**
**Model I**				
One line effect	1.00 (0.98, 1.02) 0.9896	1.00 (0.99, 1.01) 0.9438	0.99 (0.98, 1.01) 0.3491	0.99 (0.98, 1.00) 0.2044
**Model II**				
Turning point (K)	137.5	137.5	137.5	137.5
< K effect 1	0.95 (0.92, 0.98) 0.0007	0.95 (0.93, 0.98) 0.0001	0.95 (0.93, 0.97) < 0.0001	0.96 (0.94, 0.98) < 0.0001
> K effect 2	1.03 (1.01, 1.06) 0.0040	1.03 (1.01, 1.05) 0.0012	1.03 (1.01, 1.04) 0.0032	1.02 (1.01, 1.04) 0.0080
Effect 2–1	1.09 (1.04, 1.13) 0.0001	1.08 (1.05, 1.12) < 0.0001	1.08 (1.04, 1.11) < 0.0001	1.06 (1.04, 1.09) < 0.0001
LRT test	< 0.001	< 0.001	< 0.001	< 0.001

Data were presented as HR (95% CIs) P-value; Model I, linear analysis; Model II, non-linear analysis. LRT test, Logarithmic likelihood ratio test (p < 0.05 means Model II is significantly different from Model I, which indicates a non-linear relationship).

### Results of the adjusted and unadjusted Cox proportional hazard models

Table 3 displays the effect sizes [hazard ratios (HRs)] and 95% confidence intervals. The relationship between serum sodium levels and overall mortality was U-shaped. The results of the multiple regression analysis showed that in the present study, we found that CHF patients had the lowest mortality rate at serum sodium levels =137.5 mmol/L after adjusting for other covariates. Excessively high or low serum sodium levels will increase the mortality of patients with CHF. In this study, we analyzed serum sodium levels' independent impact on overall mortality (Cox proportional hazards univariate and multivariate models). Table 3 displays effect sizes [hazard ratios (HRs)] and 95% confidence intervals.

**Table 3:** Association of sodium with mortality.

**Variable**	**Crude model HR (95% CIs) *P*-value**	**Model I** **HR (95% CIs) *P*-value**	**Model II HR (95% CIs) *P*-value**
**30-day mortality**			
Sodium (mmol/L) segment 1(< 137.5)	0.96 (0.94, 0.98) 0.0001	0.95 (0.93, 0.98) < 0.0001	0.96 (0.94, 0.99) 0.0067
Sodium (mmol/L) segment 2 (≥137.5)	1.07 (1.05, 1.09) < 0.0001	1.04 (1.03, 1.06) < 0.0001	1.02 (1.00, 1.05) 0.0310
**Sodium (mmol/L)groups**			
< 135	1.59 (1.33, 1.89) < 0.0001	1.47 (1.23, 1.76) < 0.0001	1.32 (1.09, 1.59) 0.0051
≥135, < 140	Reference	Reference	Reference
≥140, < 145	1.34 (1.15, 1.56) 0.0002	1.20 (1.03, 1.40) 0.0209	1.19 (1.01, 1.40) 0.0420
≥145	2.68 (2.15, 3.35) < 0.0001	2.05 (1.64, 2.57) < 0.0001	1.46 (1.13, 1.88) 0.0032
**90-day mortality**			
Sodium (mmol/L) segment 1(< 137.5)	0.96 (0.94, 0.98) < 0.0001	0.96 (0.94, 0.98) < 0.0001	0.96 (0.94, 0.99) 0.0023
Sodium (mmol/L) segment 2 (≥137.5)	1.07 (1.05, 1.08) < 0.0001	1.04 (1.03, 1.06) < 0.0001	1.02 (1.00, 1.04) 0.0141
**Sodium (mmol/L)groups**			
< 135	1.56 (1.34, 1.80) < 0.0001	1.45 (1.25, 1.69) < 0.0001	1.28 (1.09, 1.50) 0.0031
≥135, < 140	Reference	Reference	Reference
≥140, < 145	1.33 (1.17, 1.51) < 0.0001	1.20 (1.05, 1.36) 0.0061	1.16 (1.01, 1.34) 0.0305
≥145	2.47 (2.04, 3.01) < 0.0001	1.91 (1.57, 2.32) < 0.0001	1.33 (1.07, 1.66) 0.0102
**365-day mortality**			
Sodium (mmol/L) segment 1 (< 137.5)	0.96 (0.94, 0.97) < 0.0001	0.96 (0.94, 0.97) < 0.0001	0.96 (0.94, 0.98) < 0.0001
Sodium (mmol/L) segment 2 (≥137.5)	1.06 (1.04, 1.07) < 0.0001	1.04 (1.02, 1.05) < 0.0001	1.02 (1.00, 1.03) 0.0395
**Sodium (mmol/L)groups**			
< 135	1.54 (1.36, 1.74) < 0.0001	1.44 (1.28, 1.63) < 0.0001	1.28 (1.11, 1.46) 0.0004
≥135, < 140	Reference	Reference	Reference
≥140, < 145	1.28 (1.15, 1.42) < 0.0001	1.16 (1.04, 1.29) 0.0078	1.11 (0.99, 1.25) 0.0781
≥145	2.30 (1.95, 2.73) < 0.0001	1.77 (1.49, 2.10) < 0.0001	1.29 (1.06, 1.56) 0.0101
**4-year mortality**			
Sodium (mmol/L) segment 1(< 137.5)	0.97 (0.95, 0.98) < 0.0001	0.97 (0.95, 0.98) < 0.0001	0.96 (0.95, 0.98) < 0.0001
Sodium (mmol/L) segment 2 (≥137.5)	1.06 (1.04, 1.07) < 0.0001	1.04 (1.02, 1.05) < 0.0001	1.01 (1.00, 1.03) 0.0860
**Sodium (mmol/L)groups**			
< 135	1.41 (1.27, 1.57) < 0.0001	1.34 (1.21, 1.49) < 0.0001	1.25 (1.11, 1.40) 0.0001
≥135, < 140	Reference	Reference	Reference
≥140, < 145	1.24 (1.14, 1.36) < 0.0001	1.14 (1.04, 1.24) 0.0038	1.07 (0.97, 1.18) 0.1737
≥145	2.21 (1.91, 2.56) < 0.0001	1.72 (1.48, 1.99) < 0.0001	1.24 (1.05, 1.46) 0.0100

Models 1 and 2 were derived from Cox proportional hazards regression models: model 1 covariates were adjusted for age and sex; model 2 covariates were adjusted for age; heart rate; SBP, systolic blood pressure; temperature; SPO2, pulse oxygen saturation; DBP, diastolic blood pressure; respiratory rate; albumin level; Bun, blood urea nitrogen level; sodium level; Pt, prothrombin time; platelet level; hemoglobin level; Ptt, partial thromboplastin time; hematocrit level; glucose level; potassium level; creatinine level; bicarbonate level; serum anion gap; RDW, red blood cell distribution width level; RBC, red blood cell count; WBC, white blood cells count; sex; admission type; insurance type; deficiency anemias; blood loss anemia; coagulopathy; renal failure; hypothyroidism; complicated diabetes; uncomplicated diabetes; peripheral vascular disease; hypertension; liver disease; pulmonary circulation; valvular disease; chronic pulmonary disease; and cardiac arrhythmias.

The results of the comparison of the reference groups are representative: The unadjusted model's effect size can be explained by a relationship between the mortality rate and the difference in serum sodium levels; Results recorded from the fully adjusted Cox proportional hazard model showed that lower Serum sodium levels (<137.5 mmol/L) were related to associate multiplied risk of 30, 90, 365-day, and 4-year all-cause deaths; the HRs and 95th confidence intervals were 0.96 (0.94, 0.99), 0.96 (0.94, 0.99), 0.96 (0.94, 0.98), and 0.96 (0.95, 0.98), respectively; The higher Serum sodium levels (≥137.5 mmol/L) were related to associate multiplied risk of 30, 90, 365-day, and 4-year all-cause deaths; the HRs and 95th confidence intervals were 1.02 (1.00, 1.05), 1.02 (1.00, 1.04), 1.02 (1.00, 1.03), and 1.02 (1.00, 1.03), respectively. The consecutive variables are <0.05 except for the p-value of the 4-year mortality (the group of serum sodium levels≥137.5 mmol/L) is 0.08.

When serum sodium levels <137.5 mmol/L, the effect size of 0.96 for 365-day all-cause mortality in the unadjusted model means that a difference in serum sodium levels is associated with a difference in risk of death [0.96, 95%CI (0.94, 0.98)]. In the minimum-adjusted model [0.96, 95%CI (0.94, 0.97)]. In the fully adjusted model (adjusted for all covariates presented in Table 1) [0.96, 95%CI (0.94, 0.98)].

When serum sodium levels ≥137.5 mmol/L, the effect size of 1.06 for 365-day all-cause mortality in the unadjusted model means that a difference in serum sodium levels is associated with a difference in risk of death [1.06, 95%CI (1.04, 1.07)]. In the minimum-adjusted model [1.04, 95%CI (1.02, 1.05)]. In the fully adjusted model (adjusted for all covariates presented in Table 1) [1.02, 95%CI (1.00, 1.03)].

The *P*-value for the trend of serum sodium levels with categorical variables in the fully adjusted model was consistent with the result when serum sodium levels were a continuous variable. In the sensitivity analysis, we changed serum sodium levels from a continuous variable to a categorical variable (the clinical cutoff point of serum sodium levels). Furthermore, it was discovered that the trend in effect size between groups of serum sodium levels was not uniform. The all-cause mortality findings for 30, 90 days, and 4 years were comparable to those for 365 days, as shown in Table 3, which is a strong and trustworthy indicator.

#### The results of non-linearity in serum sodium levels and all-cause mortality

**Figure 3:**
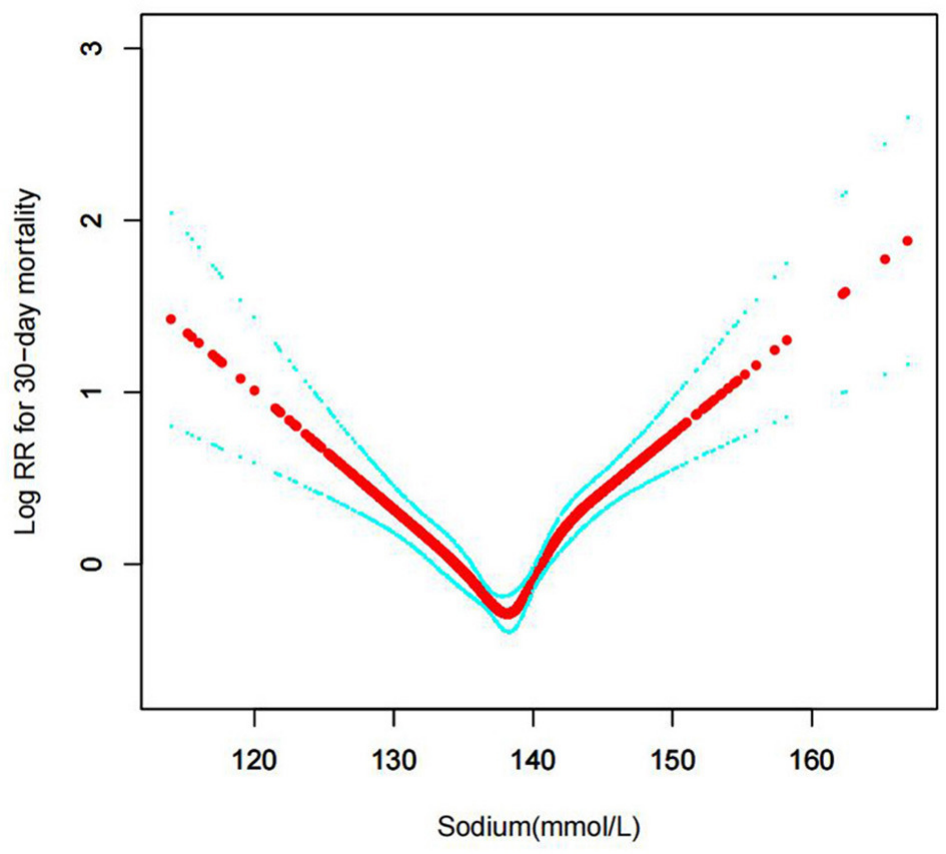
Association between serum sodium levels and 30-day all-cause mortality (Unadjusted model).

**Figure 4:**
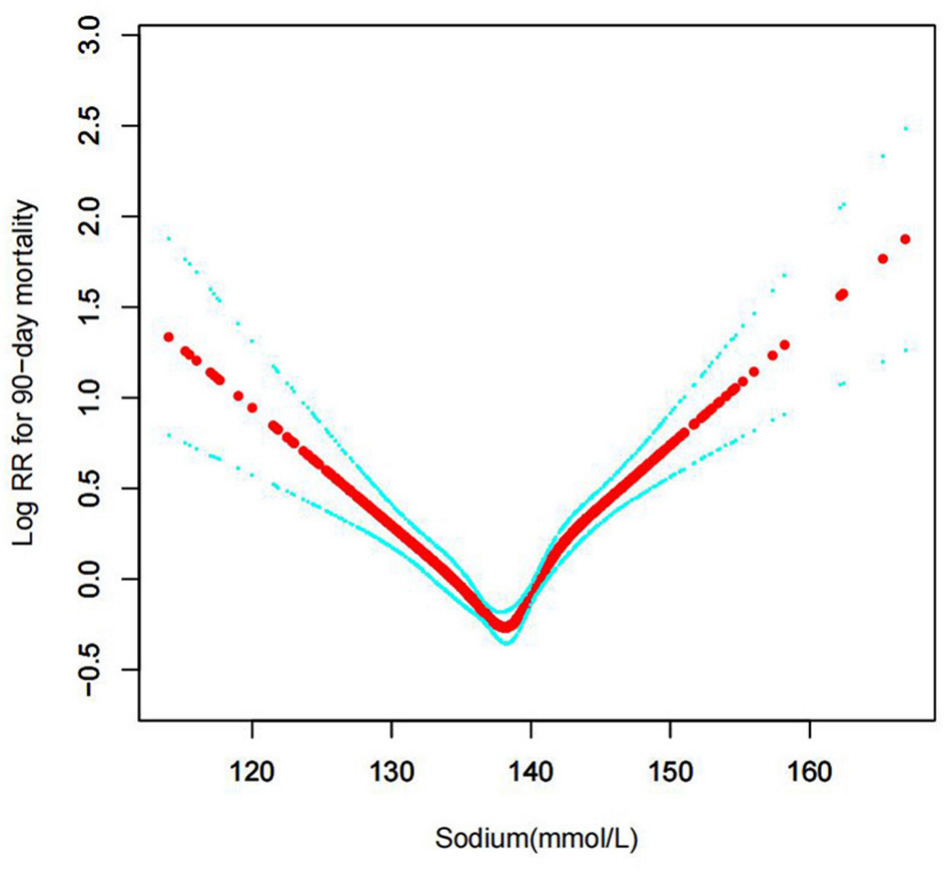
Association between serum sodium levels and 90-day all-cause mortality (Unadjusted model).

**Figure 5:**
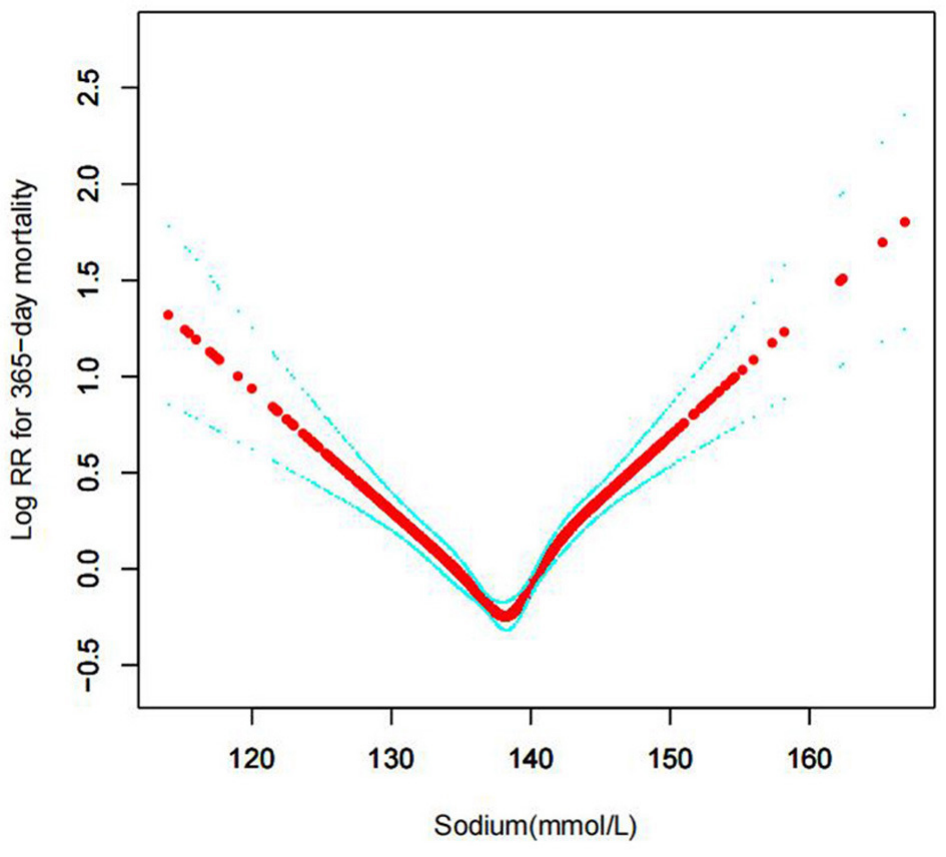
Association between serum sodium levels and 365-day all-cause mortality (Unadjusted model).

**Figure 6:**
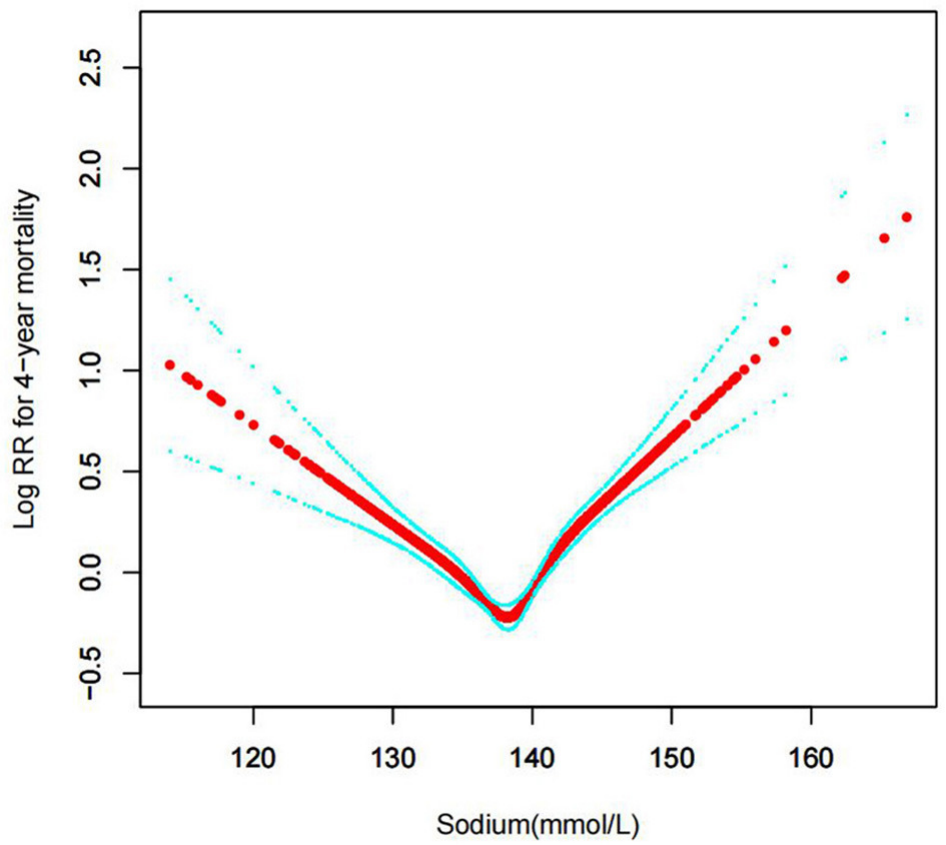
Association between serum sodium levels and 4-year all-cause mortality (Unadjusted model).

**Figure 7:**
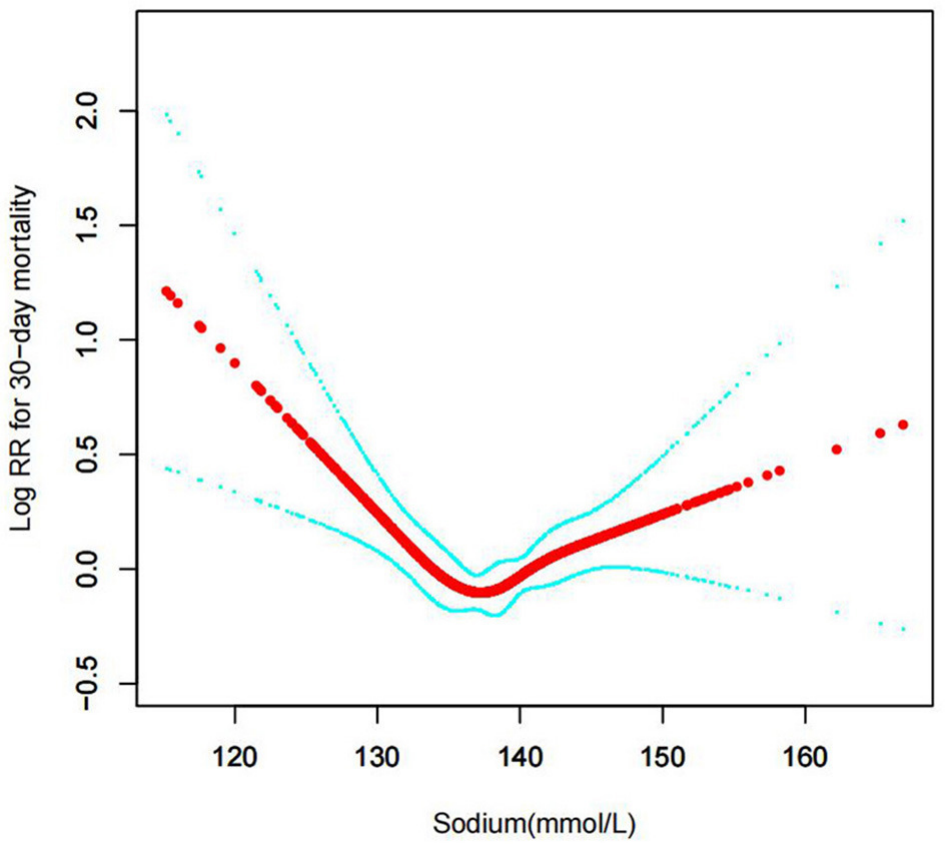
Association between serum sodium levels and 30-day all-cause mortality (After adjustment for other covariates). A generalized additive model (GAM) revealed a threshold, non-linear relationship between serum sodium levels and 30-day all-cause death. U-shaped relationships were observed after adjustment for other covariates by spline smoothing plot. The smooth curve fit between variables is shown by a solid rad line. The 95% confidence interval from the fit is represented by imaginary blue lines.

**Figure 8:**
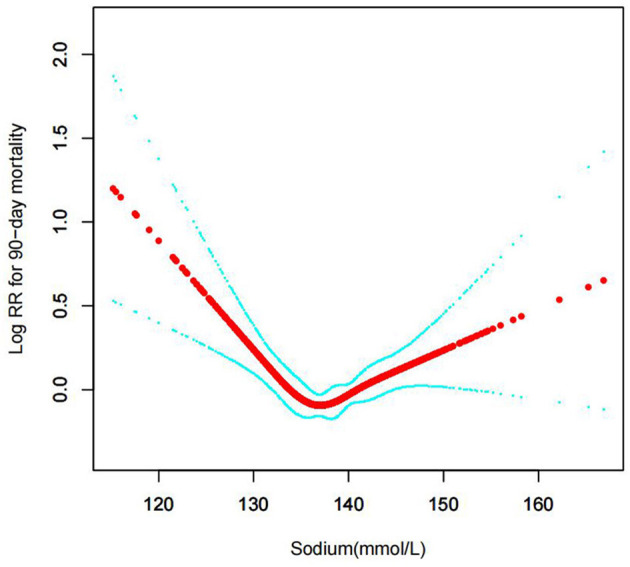
Association between serum sodium levels and 90-day all-cause mortality (After adjustment for other covariates). A generalized additive model (GAM) revealed a threshold, non-linear relationship between serum sodium levels and 90-day all-cause death. U-shaped relationships were observed after adjustment for other covariates by spline smoothing plot. The smooth curve fit between variables is shown by a solid rad line. The 95% confidence interval from the fit is represented by imaginary blue lines.

**Figure 9:**
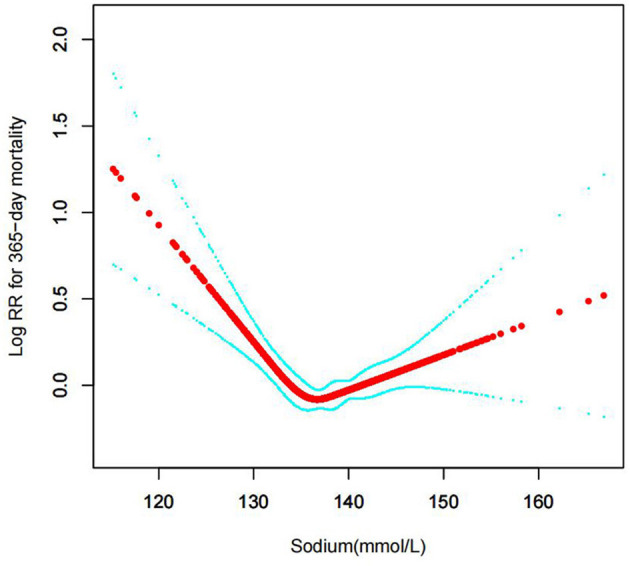
Association between serum sodium levels and 365-day all-cause mortality (After adjustment for other covariates). A generalized additive model (GAM) revealed a threshold, non-linear relationship between serum sodium levels and 365-day all-cause death. U-shaped relationships were observed after adjustment for other covariates by spline smoothing plot. The smooth curve fit between variables is shown by a solid rad line. The 95% confidence interval from the fit is represented by imaginary blue lines.

**Figure 10:**
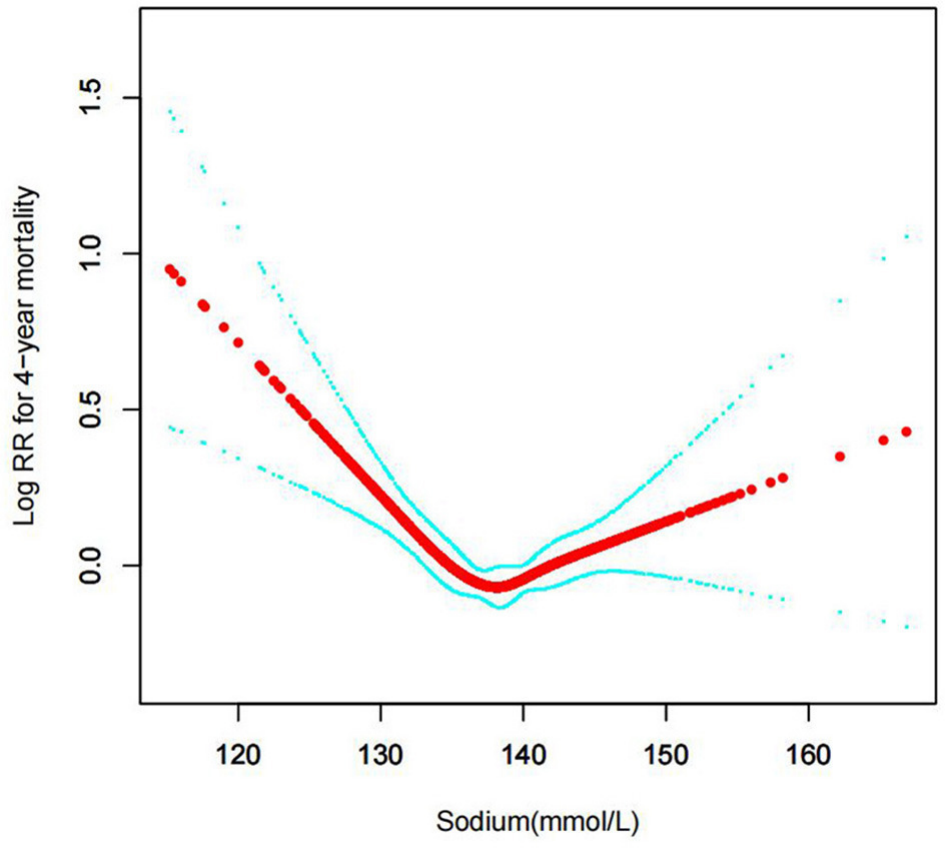
Association between serum sodium levels and 4-year all-cause mortality (After adjustment for other covariates). A generalized additive model (GAM) revealed a threshold, non-linear relationship between serum sodium levels and 4-year all-cause death. U-shaped relationships were observed after adjustment for other covariates by spline smoothing plot. The smooth curve fit between variables is shown by a solid rad line. The 95% confidence interval from the fit is represented by imaginary blue lines.

In the current study, we examined the non-linear association between serum sodium levels and fatalities from all causes within 30, 90, 365 days, and 4 years (Figures 3–6 represent the unadjusted model, and Figures 7–10 represent the fully adjusted model). The smooth curve and the result of the Cox regression model with proportional hazards using a cubic spline function suggest that the association between serum sodium and all-cause mortality is non-linear after adjusting for Gender, Admission type, Insurance, Pulmonary circulation, Peripheral vascular, Diabetes complicated, Valvular disease, Hypothyroidism, Hypertension, Renal failure, Diabetes uncomplicated, Liver disease, Cardiac arrhythmias, Coagulopathy, Chronic pulmonary, Blood loss anemia, Deficiency anemias (Figures 7–10).

The distribution of sodium levels, recognized sodium clinical ranges, and the clear u shape of the Cox proportional hazards regression model with cubic spline functions led to the classification of baseline serum sodium as <135, 135–140, 140–145, ≥145 mmol/dL. The segmentation regression model between blood sodium levels and all-cause mortality identified serum sodium levels at 137.5 mmol/L as the turning point value. The mortality of CHF patients was lowest when the serum sodium levels were 137.5 mmol/L.

#### Survival status of the patients with different admission serum sodium levels

**Figure 11:**
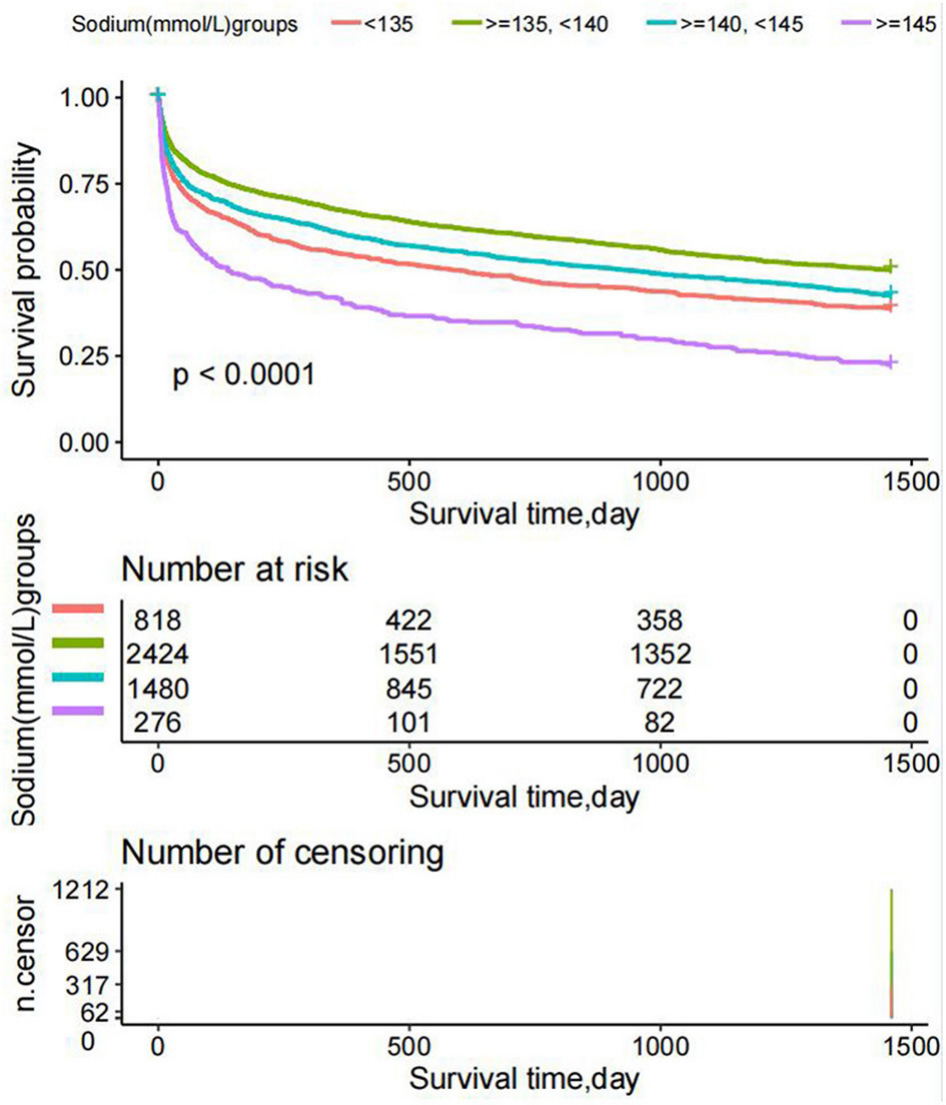
Kaplan–Meier survival curves demonstrating differences in overall survival (years).

The K-M survival curve showed that the survival time values for patients in every serum sodium group were G2>G3> G1> G4 (*P* < 0.0001) at any time within 4 years, as shown in Figure 11. The reason for the higher mortality rate in the G4 group (serum sodium levels ≥145 mmol/L) is that the patients in this group are older.

## Discussion

Congestive heart failure (CHF), characterized by sodium water retention, is the main cause of the terminal stages and death of various cardiovascular diseases. The sympathetic nervous system (SNS) and the renin-angiotensin-aldosterone system (RAAS) are intimately associated with morbidity and death with the progression of heart failure. In turn, hyponatremia encourages greater RAAS activation, starting a vicious cycle (10).

According to numerous studies, hyponatremia at admission is strongly and consistently correlated with patients' prognoses for heart failure (11–13). Results consistent with our study have been demonstrated in previous studies. On the one hand, similar findings have been observed in research examining the prognostic importance of variations in serum sodium levels in heart failure patients. In patients hospitalized with heart failure and hyponatremia, the change in serum sodium concentration over time is a significant predictive factor (5, 14). On the other hand, patients who had FHA at the time of admission but had no hyponatremia when they were discharged had a considerably worse prognosis than those who were able to keep their salt levels normal (15). In patients hospitalized for heart failure, hyponatremia at hospital admission is an independent predictor of short- and long-term death. Additionally, clinical outcomes in individuals with reduced or intact LVEF were linked to treatment-related changes in sodium levels (16).

Clinical congestive heart failure most frequently manifests as left heart failure, and the clinical features of left heart failure are mainly due to pulmonary congestion and pulmonary edema caused by left atrial failure and right ventricular failure (17). The clinical feature of right heart failure is that due to right atrial and ventricular failure caused by congestion of the veins in the systemic circulation and retention of water and sodium, the right heart frequently has functional impairment following the beginning of left heart failure, which ultimately leads to total heart failure (18). Sodium ions are very important cations in the extracellular fluid of the human body, and over 90% of the total ions in the extracellular fluid are sodium ions. In the past, textbooks have emphasized sodium restriction as a key component in treating HF patients. Data on this management style, however, is debatable (19).

Some studies have shown that the blood sodium value in patients with congestive heart failure is lower than in normal people, and the difference is significant; the worse the degree of heart function, the lower the blood sodium value, and the difference is significant; hyponatremia is common in patients hospitalized for heart failure (HF) (20–23). Additionally, it has been discovered to be a poor predictor of outcome in both outpatients and inpatients with HF. It is linked to both short- and long-term negative consequences, such as all-cause mortality (24).

A growing body of research indicates that regardless of LVEF, hyponatremia, and hypernatremia at hospital admission and discharge predict unfavorable outcomes in patients with HF (25). In comparison to other LVEF groups, HF with intermediate ejection fraction (HFrEF) patients experienced higher hyponatremia at discharge (26). As a result, in HF patients who are hospitalized, returning to normal salt levels may be the main objective. Fluid restriction and diuretics, which are broad measures that should be taken into consideration in all patients, are the most commonly used therapies for hyponatremia (27). According to the findings of the current investigation, blood sodium levels are a significant predictor of survival in people with severe chronic heart failure (28). The observation that serum sodium levels at the time of hospitalization for heart failure are related to 30-day survival raises this possibility; that treatments for hyponatremia patients that worsen or do not improve serum sodium levels may have unfavorable effects (29, 30).

The relationship between serum sodium levels and the clinical outcomes of cardiovascular events has been examined in a number of studies. Serum sodium levels were shown to have a J-shaped relationship with all-cause mortality by Patel et al. In patients with heart failure, the serum sodium level is linked to a higher risk of long-term all-cause mortality (30). Changes in serum sodium levels have previously been shown to be a significant predictor of outcomes. According to Avc et al. hyponatremia is common in Turkish patients hospitalized for increasing heart failure. An independent and significant risk factor for increased mortality in this population was low serum salt concentration (23). According to Shorr et al.'s research, chronic hyponatremia raises the probability of unfavorable outcomes following discharge, which raises the possibility that hyponatremia may play a role in the pathophysiology of HF (31).

In conclusion, serum sodium levels, as a cheap, cost-effective, and easily available blood test index, can be used to judge the degree of heart failure and predict the risk of death in patients with congestive heart failure. Here we discuss the relationship between serum sodium levels and all-cause mortality. We discuss the relationship between serum sodium levels and all-cause mortality and provide a reference for clinical diagnosis and treatment according to the serum sodium levels, the degree of heart failure, and appropriate supplementation or reduction of sodium.

The significance of this clinical study is as follows: (1) We found curved relationships between serum sodium levels and 30, 90, 365-day, and 4-year all-cause mortality among CHF patients receiving intensive care; (2) The research success can aid in the development of preventative, predictive, diagnostic, and prognostic serum sodium level models for CHF patients over the short, medium, and long term. (3) One potential unique aspect of our manuscript is the longitudinal analysis, which refers to an investigation where participants and serum sodium levels are collected at multiple different follow-up timestamps (30, 90, 365-day, and 4-year). (4) Compared with previous studies, we not only studied the long-term mortality over 4 years but also used cubic splines to make the results more intuitive.

Several strengths in our study: (1) The Mimic-III database is a comprehensive, openly accessible repository with trustworthy data, a wide range of covariables, and adequate confounding factor modification. (2) Because the study is non-linear, according to the smooth curve fitting diagram that was employed, the outcomes were steady. Our results are further validated by the K-M survival curve approach, which is beneficial for future study discoveries. (3) No patients were harmed or violated in the course of this investigation. (4) Our follow-up period was longer compared to earlier research on the connection between serum sodium levels and heart failure.

A few limitations in our study: (1) Because CHF was the research subject, there are some restrictions on the generalizability and extrapolation of this study, and it does not apply to individuals with other conditions. (2) The serum sodium level was initially measured when the patient was admitted to the intensive care unit (ICU), so the results can be skewed. (3) We only investigated the impact of the serum sodium levels on those patients. To assess this disease deeply, we will analyse those risk factors affecting the basic conditions in the future study. (4) Our research was unable to elucidate the underlying mechanisms of serum sodium levels and all-cause mortality, which requires further study. (5) As the data we extracted were baseline indicators for patients on the first day of icu admission, there was no way to obtain information on patients' use of diuretics, which are often a common cause of hyponatraemia, and due to the limitations of the MIMICIII database plasma B-type natriuretic peptide (BNP) or N-terminal pro-BNP levels had too many missing values, so they were not taken into account in our study.

## Conclusion

Our data show that after controlling for other factors, serum sodium levels were u-shaped for all-cause mortality. In individuals with CHF, elevated serum sodium levels are linked to an elevated risk of short-, medium-, and long-term all-cause mortality. The chance of dying from congestive heart failure (CHF) is closely correlated with elevated or decreased blood sodium levels, which may also be a new risk factor for cardiovascular disease.

## Data availability statement

Publicly available datasets were analyzed in this study. This data can be found at: https://mimic.mit.edu/.

## Ethics statement

The Massachusetts Institute of Technology (Cambridge, MA) and Beth Israel Deaconess Medical Center (Boston, MA) approved the database's establishment, and consent was acquired for the first data gathering. Hence, this research was exempt from the ethical approval statement and the necessity of informed consent.

## Author contributions

SP, JP, and LY were in charge of the research's overall execution and manuscript writing. WK was in charge of analyzing the data. All authors contributed to the article and approved the submitted version.
